# A Manual of Toxicology

**Published:** 1874-06-01

**Authors:** 


A Manual of Toxicology. By John J. Ruse, M. D., Prof. of Medical Jurisprudence and Toxicology, in the University of Pennsylvania, etc. Philadelphia: Lippincott & Co., March, 1874.

This work is from the pen of the editor of the seventh American edition of Taylor's Manual of Medical Jurisprudence, which was published in September, 1873, and might really be considered the eighth edition of that valuable book. While the size, as well as value, of Taylor's "Manual" has been much enhanced by copious contributions by Prof. Ruse, the latter, in writing his own work, used essentially the same material. Though it is difficult to see the necessity of publishing, within a period of six months, two almost identical works, and giving the credit of authorship to different persons, it must be admitted that Ruse's "Manual" has features which recommend it preferably to Taylor's, as a text-book for students.

It is but little over half the size of Taylor's work, and containing almost as much information, it follows that the language is more concise and correspondingly plainer. In matter it is fully abreast of the times: articles of recent introduction into common use being fully treated of. Spectral analysis is not treated of at all, though the author regards it as an exceedingly valuable corroborative means of evidence. He justly holds that, in a case of alleged poisoning, it is not safe to rest the evidence solely upon the spectral demonstration of the supposed agent, to the exclusion of chemical tests. The work is one of the best in the English language.

W. E. Q.

---

MORTALITY TABLES OF CHICAGO FOR THE TWO WEEKS ENDING MAY 9TH, 1874.—Angina Pectoris, 1; Appoplexy, 3; Brain, Congestion of, 6; Brain, Inflammation of, 2; Brain, Softning of, 1; Bronchitis, 3; Cancer, 1; Cancer of Liver, 1; Cancer of Stomach, 3; Cancer of Uterus, 2; Cellulitis, 1; Consumption, 25; Convulsions, 29; Croup, 3; Debility, 4; Delirium Tremens, 1; Diphtheria, 2; Dropsy, 4; Dysentery, 2; Enteritis, 6; Endocarditis, 2; Fever, Intermittent, 1; Fever, Puerperal, 5; Fever, Scarlet, 7; Fever, Remittent, 2; Gastritis, 1; Gastroenteritis, 3; Heart Disease, 4; Hydrocephalus, 3; Hydrophobia, 1; Inanition, 4; Kidneys, Bright's Disease of, 2; Laryngitis, 3; Liver, Cirrhosis of, 1; Liver, Inflammation of, 1; Lungs, Congestion of, 7; Lungs, Hæmorrhage of, 1; Malformation, 2, Measels, 1; Meningitis, 8; Meningitis, Cerebro Spinal, 7; Old Age, 2; Peritonitis, 4; Paralysis, 1; Parotitis, 1; Pleurisy, 3; Pneumonia, 14; Pneumonia, Typhoid, 5; Pyemia, 1; Septicemia, 1; Small Pox, 8; Stomach, Ulceration of, 1; Tabes Mesenterica, 7; Teething, 3; Tetanus, 2; Whooping Cough, 6; Accidents, 5.

AGES.—Under 1 year, 71; from 1 to 2 years, 15; from 2 to 3 years, 8; from 3 to 4 years, 6; from 4 to 5 years, 9; from 5 to 10 years, 13; from 10 to 20 years, 11; from 20 to 30 years, 20; from 30 to 40 years, 35; from 40 to 50 years, 19; from 50 to 60 years, 17; from 60 to 70 years, 10; from 70 to 80 years, 5, from 80 to 90 years, 1. Total, 241.

---

HYPODERMIC INJECTIONS OF PHENIC ACID IN INTERMITTENT FEVER.—Dr. Barberis mentions two cases of intermittent fever in which recovery followed upon subcutaneous injections of phenic acid. He hardly, however, feels warranted in drawing any conclusions at present, but invites the repetition of the experiment. The two cases which he has treated in this manner were quite dissimilar; in the first, there was a periodical fever of long standing and of miasmatic origin, with enlargement of the spleen and leucæmia. In the second case, the fever was of rheumatic origin with an abnormal course, being at first irregular, but afterwards approaching the continued type. In both cases quinine had been administered without success.

The injections of phenic acid are well tolerated and do not give rise to any local disturbance. The solution should be from fifteen to twenty parts in one hundred. The quantity of the acid which one may inject varies from one-sixth to one grain (one-hundredth to five-hundredths centigrammes.)—*Journal de Therapeutique and Gazzetta delle Clinche.*

